# Current Knowledge on the Use of Computational Toxicology in Hazard Assessment of Metallic Engineered Nanomaterials

**DOI:** 10.3390/ijms18071504

**Published:** 2017-07-12

**Authors:** Guangchao Chen, Willie Peijnenburg, Yinlong Xiao, Martina G. Vijver

**Affiliations:** 1 Institute of Environmental Sciences, Leiden University, 2300 RA Leiden, The Netherlands; willie.peijnenburg@rivm.nl (W.P.); xiao@cml.leidenuniv.nl (Y.X.); vijver@cml.leidenuniv.nl (M.G.V.); 2 Centre for Safety of Substances and Products, National Institute of Public Health and the Environment (RIVM), Bilthoven, 3720 BA Bilthoven, The Netherlands

**Keywords:** computational toxicology, hazard assessment, metallic engineered nanomaterials, (quantitative) structure–activity relationships, species sensitivity distributions

## Abstract

As listed by the European Chemicals Agency, the three elements in evaluating the hazards of engineered nanomaterials (ENMs) include the integration and evaluation of toxicity data, categorization and labeling of ENMs, and derivation of hazard threshold levels for human health and the environment. Assessing the hazards of ENMs solely based on laboratory tests is time-consuming, resource intensive, and constrained by ethical considerations. The adoption of computational toxicology into this task has recently become a priority. Alternative approaches such as (quantitative) structure–activity relationships ((Q)SAR) and read-across are of significant help in predicting nanotoxicity and filling data gaps, and in classifying the hazards of ENMs to individual species. Thereupon, the species sensitivity distribution (SSD) approach is able to serve the establishment of ENM hazard thresholds sufficiently protecting the ecosystem. This article critically reviews the current knowledge on the development of in silico models in predicting and classifying the hazard of metallic ENMs, and the development of SSDs for metallic ENMs. Further discussion includes the significance of well-curated experimental datasets and the interpretation of toxicity mechanisms of metallic ENMs based on reported models. An outlook is also given on future directions of research in this frontier.

## 1. Introduction

Nanotechnology has been identified as a key-enabling technology by the European Commission [1]. It is seen as one of the sectors bringing economic benefit and jobs. The extensive use of engineered nanomaterials (ENMs), however, has raised concerns about their possible effects on human health and their environmental burden [2]. Laboratory observations on some potentially harmful effects of ENMs have in some cases overshadowed the immense promise of these materials and their nanotechnology applications [3,4]. As concluded by the EU NanoSafety Cluster, the real concern, rather than fragmentary observations on some hazards of exposure to ENMs, is the lack of systematic studies on adverse effects or exposure to ENMs [5]. Since experimental testing is significantly constrained by time, financial burden, and ethical considerations (such as the principles of replacement, reduction, and refinement of animal testing, i.e., the 3Rs), the use of computational tools as alternative or compensation is expected to provide an efficient and inexpensive way of meeting the data requirements for the purpose of managing ENM risks [6]. Computational toxicology is seen as a potential tool to reduce the tension caused by the lag of evaluating nanosafety in respect to the rapid development of nanotechnology and nano-related innovation. Computational toxicology is emerging as a tool with active development and great potential [7], and is able to create predictive power in the field of toxicology with the aid of modern computing and information technology [8,9].

Computational tools combined with powerful data-mining technologies, have been proposed to model chemical properties of soluble chemicals [10,11,12], biological activities [6], and species sensitivity distributions (SSDs) [13]. The successful application of computational toxicology for soluble chemicals has promoted the expansion of these in silico approaches into the field of hazard identification of ENMs. Reliable computational tools can contribute to the supplementation of data for the gathering and evaluation of information as the first step of ENM hazard assessment recommended by the European Chemicals Agency (ECHA); or assist in the second step of hazard assessment (categorization and labeling of ENMs) by directly classifying ENMs into groups of different hazard [14]. For ENMs that meet the criteria of any of the hazard categories listed by ECHA, the use of the SSD method is helpful for deriving hazard threshold levels, e.g., predicted no effect concentration for the ecosystem as the last step of the ENM hazard assessment [14]. The information obtained on the basis of these steps is crucial for the qualitative risk characterization of ENMs; the structural characteristics that are identified by computational tools as governing toxicity may provide guidance for the safe-by-design of ENMs. This article reviews the current knowledge on the use of computational toxicology in the hazard assessment of metallic ENMs. A literature search was performed by means of an Advanced Search in the Web of Science^TM^ Core Collection on 22 February 2017, manually supplemented with relevant publications not included in the search records. The studied metallic ENMs in this review are restricted to metal ENMs and metal oxide ENMs. Other types of ENMs such as quantum dots are out of the scope of the research. To provide an overview of the key issues in this field, the following aspects are discussed in the review: (i) the state-of-the-art of the development of nano-(Q)SARs and read-across, and of the development of SSDs for metallic ENMs; (ii) the availability of laboratory-derived data for ENM-related modeling; (iii) the interpretation of toxicity mechanisms of metallic ENMs based on developed models; and (iv) an outlook on future directions of research in this frontier. This information will be of benefit to the researchers working in the field of ENM-related modeling and also to product developers aiming to introduce safe and environmentally benign ENMs to the market. The discussion on environmental risks of metallic ENMs will serve regulatory purposes.

## 2. State-of-the-Art of In Silico Models Serving Hazard Assessment of ENMs

In this part, the state-of-the-art of both the development of nano-(Q)SARs and read-across, and the development of SSDs for metallic ENMs are discussed. The reported models are reviewed and summarized in Table 1 and Table 2. For the studies of nano-(Q)SARs and read-across, the employed descriptors (measured or calculated), number of ENMs in the datasets, tested organisms, and data resource are analyzed. In order to extract information for the safe-by-design of ENMs on the basis of derived models, the role of different factors (represented by descriptors in the models) in affecting nanotoxicity is analyzed. With respect to the development of SSDs for metallic ENMs, the types of metallic ENMs, hazards thresholds as represented by the 5th percentile of SSDs (HC5), number of species in SSDs, and associated environmental compartments are described. The estimated HC5s (aquatic) of different metallic ENMs are compared. Meanwhile, relevant risks reported for different ENMs in different environmental compartments are also presented.

### 2.1. Development of (Q)SARs and Read-Across Models for Metallic ENMs

In general, the derivation of predictive models includes the steps of data assembling, structure characterization, model construction, model evaluation, and lastly interpretation of mechanisms [6]. Based on distinct mathematical approaches, the developed models may be designed to offer quantitative estimates for the hazardous effects elicited by metallic ENMs, or to contribute to the categorization and labeling of ENMs of interest. The analysis of reported models in Table 1 shows that both types of models have been introduced for assessing the toxicity of metallic ENMs to biota. More than half of the studies (14 out of 22) originating from the literature review focused on the numerical prediction of ENM toxicity. Interestingly, the prediction of biological effects of metallic ENMs to *Escherichia coli* or to different cell lines seems to be of special research interest, as only three studies reported predictive models for species of other types [15,16,17]. Given the current advances, it seems that categorical prediction of ENM toxicity could potentially serve relevant risk assessment targeting a relatively broader spectrum of species. For most of the in silico models, the datasets used are relatively small, which probably poses major limitation on their potential applicability.

The frequently appearing descriptors in the models may encode important messages on ENM characteristics dominating relevant biological activities. This kind of messages benefits both the hazard assessment and the safe-by-design of ENMs. Thus, the presented descriptors in existing models are summarized (see Table 1) and analyzed to discuss the role of different factors in influencing nanotoxicity. As for studies introducing multiple models or incorporating a big variety of descriptors, only main factors as highlighted by the authors are considered to avoid the impact of possible accidental correlations. The analysis show that some of the statistical models comprise merely theoretical descriptors; meanwhile, the experimental parameters such as zeta potential, concentration of ENMs, aggregation parameter, size of the particles in media etc. are also found to be incorporated into other models. Subsequently, these descriptors are roughly labeled as belonging to one of three general types for further analysis: the intrinsic properties of the metal or metal oxide, the nano-specific characteristics of ENMs, and the dynamic changes of ENMs in media. The factors affecting ENM toxicity are further discussed below.

(i) Descriptors regarding the intrinsic properties of metal (oxide):
  - Surface catalytic properties and redox modifications related factors include: Wigner–Seitz radius, mass density, band gap energy, overlap of conduction band energy levels with the cellular redox potential, conduction band energy, average of the alpha and beta LUMO (lowest unoccupied molecular orbital) energies of the metal oxide, accessible surface area, absolute electronegativity of the metal and the metal oxide, aligned electronegativity, electronegativity, Mulliken’s electronegativity of the cluster, S_2_ (SiRMS-derived number of oxygen’s atoms in a molecule, which was described by their electronegativity), S_3_ (tri-atomic fragments[Me]-[O]-[Me], which were encoded by SiRMS-derived descriptors, encoding electronegativity), and metal electronegativity.
  - Characteristics related to the capability of ion and electron detachment and the activity of ions include: covalent index, cation polarizing power, atomization energy, metal oxide ionization energy, ionic index of metal cation, enthalpy of formation of metal oxide nanocluster representing a fragment of the surface, cationic charge, enthalpy of formation of a gaseous cation, charge of the metal cation corresponding to a given oxide, solubility, polarizability, molar refractivity, and polarization force.
(ii) The nano-specific descriptors employed in the developed models include:
  - The size of ENMs; and
  - Parameters characterizing the surface chemistry of ENMs, e.g., hydrophobicity of surface coating chemicals, surface-area-to-volume ratio, surface coating and charge, surface area, polar surface area.
(iii) The parameters indicating the dynamic changes of ENMs in media include:
  - Zeta potential;
  - Concentration of ENMs; and
  - Descriptors representing the dispersion and aggregation of ENMs in media, e.g., aggregation parameter, size in DMEM (Dulbecco’s Modified Eagle’s Medium), relaxivity (representing ENM magnetic properties), size in phosphate buffered saline, size in water, aggregation size.

**Figure 1:**
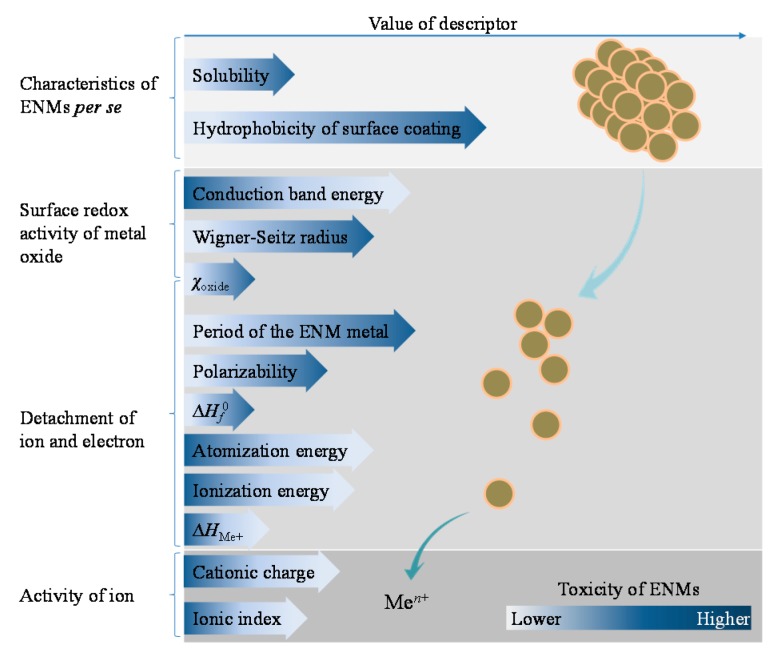
Generalization of the role of different factors in affecting the toxicity of metallic ENMs based on the state-of-the-art of nano-(Q)SARs and read-across models for ENMs. Me*^n^*^+^ represents the released ions from ENMs; ∆*H_f_*^0^ is the enthalpy of formation of metal oxide nanocluster representing a fragment of the surface; ∆*H*_Me+_ is the enthalpy of formation of a gaseous cation having the same oxidation state as that in the metal oxide structure; and χ_cation_ represents the electronegativity of the metal oxide.

Extraction of the general dependency of nanotoxicity on different factors may be of potential help for designing safe and environmentally benign ENMs. This kind of message could be derived from the quantitative models for ENMs. Descriptors reported without explicit equations of predictive models cannot serve this purpose. Despite the fact that various types of descriptors have been used in different in silico models, only a limited number of these parameters exhibited an explicit and unambiguous role in ENM-induced toxicity. The identified descriptors are roughly concluded here as concerning four aspects of the materials: the characteristics of ENMs per se, surface redox activity of metal oxides, ease of ion and electron detachment, and activity of the ion detached (see Figure 1). Some of the computational parameters may refer to multiply processes involved in the adverse effects triggered by metallic ENMs.

Hydrophobicity of ENM surface coatings and solubility of ENMs are shown to positively correlate with observed nanotoxicity. Other factors playing the same role in affecting nanotoxicity include the Wigner–Seitz radius and the electronegativity of metal oxides (χ_oxide_), which reflect the surface redox activity of the metal or metal oxide; and the period in the periodic table of the ENM core metal, polarizability, and enthalpy of formation of metal oxide nanoclusters representing a fragment of the surface (∆*H*_f_^0^), which indicates the ease of detachment of ions and electrons from ENMs. The Wigner–Seitz radius describes the available fraction of molecules on the surface of a nanocluster [33]. The χ_oxide_ characterizes the ability of atoms of metal oxides to attract electrons that contribute to the surface redox activities, and also relates to the leaching of ions from the surface of metal oxides [22]. The period of the ENM metal represents information of atomic radii of the metal which is also associated with polarizability [40].

On the other hand, the toxicity of ENMs tends to decrease with increased conduction band energy, atomization energy, ionization energy, ∆*H*_Me+_ (enthalpy of formation of a gaseous cation having the same oxidation state as the metal in the metal oxide structure), cationic charge, and ionic index. Zhang et al. [19] have evidenced the strong correlation between the toxicity of Co_3_O_4_, Cr_2_O_3_, Ni_2_O_3_, Mn_2_O_3_, and CoO ENMs and the overlap of ENMs’ conduction band energy with the cellular redox potential (−4.12 to −4.84 eV). The studied ENMs with conduction band energy out of the range failed to exhibit pro-oxidative and oxidative stress effects, with two exceptions ZnO and CuO ENMs. The exceptions could be explained by their relatively high solubility [19]. Decreasing atomization energy attributes to the decrease of the stability of metal oxides and corresponding increase of reactivity [18]. Ionization energy reflects the required amount of energy to remove the most loosely bound electron, a lower ionization energy thus indicates the easier detachment of electrons from the metal oxides [41]. ∆*H*_Me+_ describes the dissolution of ENMs without oxidation or reduction of ions, and the redox properties of metal oxides [28]. Cationic charge was also found to be an important parameter in nano-QSARs [31]. Cations (Me*^n^*^+^) with smaller charges are considered more energetically favorable than cations of larger charges, which explains why the toxicity of metal oxides decreases in the order of Me^2+^ > Me^3+^ > Me^4+^ [28]. The ionic index of cations is associated with the affinity of metal ions for water molecules (measured by the hydration enthalpy); a lower hydration enthalpy means greater transport of metal ions across cellular membranes [21]. Notably, even though most of the employed descriptors characterize the intrinsic properties of the metal or metal oxides, several factors related to the characteristics of ENMs per se were also identified as affecting toxicity.

However, the role of some factors as concluded from developed models yielded conflicting results compared with experimental observations. For instance, the smooth muscle apoptosis (SMA) was modeled by means of the core material (*I*_Fe3O4_), surface coating (*I*_dextran_), and surface charge (*I*_surf.chg_) of ENMs [26], and can be expressed as:

(1) $$SMA=2.26\left(\pm 0.72\right)-10.73\left(\pm 1.05\right)\times I_{{\mathrm{Fe}}_{2}O_{3}}-5.57\left(\pm 0.98\right)\times I_{\mathrm{dextran}}-3.53\left(\pm 0.54\right)\times I_{\mathrm{surf}.\mathrm{chg}}$$

Therefore, based on this model, it is obvious that a lower surface charge will result in higher apoptosis of smooth muscle cells. This, however, does not agree with some previous findings [42,43,44]. Reportedly, the more negative citrate-Ag ENMs were the least toxic to gram-positive *bacillus*, whereas the positively charged Ag ENMs showed the strongest toxicity [43]. For Au ENMs, both the positively and negatively surface-charged Au ENMs were found to induce significant cellular mitochondrial stress other than the Au ENMs with neutral surface charge [44]. Another study of Asati et al. [42] indicated that the surface charge of cerium oxide ENMs distinctly affects the internalization of ENMs by different cells, and the subsequent internal localization in cells which ultimately leads to the different toxicity profiles reported for cerium oxide ENMs. Meanwhile, the roles of some employed descriptors also conflict within or between independent studies. One example is the size of ENMs. The studies of both Luan et al. [23] and Kleandrova et al. [16] reported the diminution of ENM toxicity as a result of increasing ENM size. By contrast, based on the model developed by Liu et al. [18], a larger size of ENMs was shown to lead to higher nanotoxicity. It was explained that within the narrow domain of the dataset (8–19 nm), toxicity may increase with increased primary size of ENMs. A linear model developed by Papa et al. [30] also showed increased release of lactate dehydrogenase with the increment of the size of TiO_2_ and ZnO ENMs (ranging from 20 to 70 nm). In addition, the particle size in phosphate buffered saline (PBS) and in water, indicating the aggregation behavior of ENMs in media, contributes oppositely to nanotoxicity as summarized from the models developed [30].

### 2.2. Development of SSDs for Metal-Based ENMs

SSDs are commonly used for estimating the maximum acceptable concentrations of chemicals in environmental risk assessment [13]. Following its successful application to soluble chemicals, the SSD approach nowadays is also employed to rank the sensitivity of species to metallic ENMs (Table 2). The state-of-the-art of the development of SSDs for metallic ENMs shows that Ag, Al_2_O_3_, Au, CeO_2_, Cu, CuO, FeO_x_, Silica, TiO_2_, and ZnO ENMs have been commonly assessed for their adverse effects across different taxonomic groups. Compared to the diversity of ENMs involved in nano-(Q)SARs, the number of ENMs covered in SSD-related studies seems very limited. This may be because most of the derived SSDs grouped the materials solely based on their types (core material) without considering other structural characteristics. Thus, data of different ENMs with the same core was merged into the information of merely one type of ENMs. The exception is that, in the study of Garner et al. [45], separate SSDs were presented for uncoated Ag and polyvinylpyrrolidone (PVP)-coated Ag ENMs. In the study of Chen et al. (2017), separate SSDs for metallic ENMs were derived considering different ENM characteristics, experimental conditions, and toxicity endpoints. The limited variation in types of ENMs included in the SSDs is mostly due to the insufficient number of data of other type ENMs originated from experimental assays.

Nevertheless, the kinds of ENMs studied in the development of SSDs are indeed among the types that are largely found in the applications and products on the market. According to the study of Keller and Lazareva [59], the 10 major ENMs (production of >100 t/year) used within the global economy are: Ag, Al_2_O_3_, CeO_2_, Cu, Fe, SiO_2_, TiO_2_, and ZnO ENMs, carbon nanotubes, and nanoclays. An estimate of Bondarenko et al. [3] on the annual production of ENMs showed an order with regard of production volume, from high to low, of SiO_2_ (5500 t/year), TiO_2_ (3000 t/year), ZnO (550 t/year) ENMs, carbon nanotubes (300 t/year), FeO_x_ (55 t/year), CeO_x_ (55 t/year), AlO_x_ (55 t/year), Ag ENMs (55 t/year), quantum dots (0.6 t/year), and fullerenes (0.6 t/year). Therefore, it seems like it is possible to perform safety evaluation of all the metallic ENMs that are produced in high amounts. Among these ENMs, Ag ENMs have relatively gained most research attention. Chen et al. (2017) reported that Ag ENMs have been tested on the highest number of species considering the available data on LC50, EC50, LOEC, and NOEC. This enabled the development of SSDs for Ag ENMs separated by the different key factors. Further studies, ideally, should focus on other types of ENMs for the comprehensive evaluation of nanosafety. Meanwhile, besides the aquatic hazards of metallic ENMs, the potential risks brought by ENMs in other environmental compartments (e.g., air, soil) should also be considered. The implementation of these research needs however strongly depends on the quality of laboratory derived raw data. The increase of the quality of experimental data combined with robust uncertainty quantification will contribute to the improvement of the quality of SSDs.

**Figure 2:**
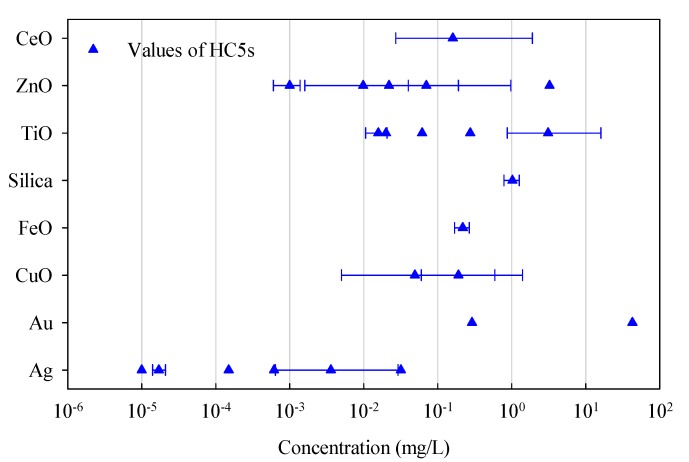
Estimated HC5s from SSDs (aquatic) for different types of ENMs. The relevant confidence intervals are also given (if available in the original publications).

The HC5s derived from the SSDs developed for different ENMs are compared as depicted in Figure 2. The HC5 values from Chen et al. (2017) were taken from the SSDs of ungrouped Ag, CuO, TiO_2_, and ZnO ENMs based on LC50 data and in case of CeO_2_ ENMs based on EC50 data for comparison. As observed, Ag, TiO_2_, and ZnO ENMs have relatively more estimates from the studies, which however also yielded much wider ranges of the reported HC5 values. The range of the HC5s of Ag ENMs indicts a higher potential of toxic impacts of the material on the environment compared with that of the ZnO and TiO_2_ ENMs. The HC5 values of silica and FeO_x_ ENMs are significantly higher than those of Ag ENMs. The median HC5 values of Au ENMs also reveal their mild toxicity compared with the toxicity of Ag ENMs. However, without the quantification of uncertainty, it is hard to conclude whether the difference is significant.

Additionally, a few studies have also presented the risk qualifications for metal-based ENMs along with the development of relevant SSDs, including Ag, Au, FeO_x_, silica, TiO_2_, and ZnO ENMs. For Ag ENMs, despite the estimated risks in surface water being shown by Haulik et al. [56] to be below 0.001 (predicted environmental concentration divided by the HC5), the studies of both Gottschalk et al. [57] and Coll et al. [49] have reported significantly higher risk probabilities of, respectively, 0.7 and 0.038, which necessitates these materials to be studied in more depth with the highest priority. Risk coefficients of Ag ENMs in soil are calculated to be always <0.01 [49,57]. The risk coefficient of Ag ENMs in sewage treatment effluent is however as high as 39.7 [57]. Risk characterizations of TiO_2_ ENMs in surface water and soil show that risks are relatively low in all studies except for the estimates reported by Coll et al. [49] as being 0.03 and 0.013, respectively; the risk coefficient of TiO_2_ ENMs in sewage treatment effluent is also relatively high (18.7). A marginal risk of ZnO ENMs in surface water (0.09) was indicted [49], whereas the risk coefficient of ZnO ENMs is again substantially higher (1.1) with respect to sewage treatment effluents [57]. For Au, FeO_x_, and silica ENMs, the derived risk probabilities are very low [47,50,51]. In short, marginal risks are reported for Ag, TiO_2_, and ZnO ENMs in surface water, and for TiO_2_ ENMs in soil, while high environmental risks were identified for Ag, TiO_2_, and ZnO ENMs in sewage treatment effluent.

## 3. The Struggle of Data Availability

Unexpectedly, in spite of the constantly increasing number of scientific resources from diverse nanosafety programs, only a relatively small number of datasets (Table 1), such as those published by Puzyn et al. [28] and Gajewicz et al. [22], are found to be repeatedly used in different modeling studies [22,27,28,31,32,33,34,36,60]. The data points used for developing SSDs are also very limited as shown in Table 2. This leads to doubts about the suitability of existing nanotoxicity data in developing models for ENMs. As explained, data scarcity may result from data incompleteness and from inconsistency in reporting the characteristics of ENMs and relevant experimental information by independent studies. This in turn leads to the difficulty of comprehensively characterizing ENM structures for performing modeling and to the difficulty of separating ENMs according to different ENM characteristics or experimental conditions [15,58]. In this context, availability of the vast majority of existing nanotoxicity data is greatly reduced and the use of this information in developing computational models for ENMs is severely prevented.

With limited available data on nanotoxicity, the developed models mostly incorporate descriptors representing only the ENM core, an approach that can also be used in the case of their corresponding bulk materials. As for further development of in silico models for ENMs, the ideal situation is to also involve comprehensive information on many of the other characteristics of ENMs such as surface chemistry, shape, dimensional aspects, crystallinity etc. for the better prediction and explanation of the biological activities of metallic ENMs [15]. The use of parameters only characterizing ENM cores in models is by far not sufficient to address nano-specific toxicity in contrast with their bulk counterparts and to distinguish the structural differences of distinct ENMs with the same core. This requires a well-defined format for reporting the observed nanotoxicity, the experimental conditions, and the used ENMs. Thoroughly curated datasets of nanotoxicity are essential for modelers to carry out further researches. Therefore, here, we propose that a report of ENM toxicity for this specific purpose should properly describe at least the following information:

(i) Details of the tested organisms, e.g., taxonomic categorization, name of species, exposure route, life-stage or bacterial strain (for bacteria);
(ii) Conditions of the performed experiments, e.g., test guideline used (if available) and possible modifications of the test guideline, preparation of test medium, composition of the exposure medium, media pH, light condition, and time-dependent medium stability;
(iii) Information on the specific toxicity endpoints, e.g., observed biological effects, type of endpoint, experimental value of toxicity endpoint, and unit in which the endpoint is expressed; and
(iv) Characteristics of the ENMs tested, e.g., type of ENMs, composition of core, distribution of particle size, surface coating, purity, crystallinity, surface area, surface charge, shape, agglomerate size and material zeta potential in media, stability in test medium.

## 4. Profiling Nanotoxicity on the Basis of In Silico Models

The development of in silico models also enabled the identification of factors of importance (represented by different descriptors) in affecting the toxicity of metallic ENMs. The hydrophobicity of surface coatings and surface charge of ENMs are shown to play an important role in determining nanotoxicity. These descriptors characterize the surface chemistry of metallic ENMs and are seen as nano-specific descriptors. The experimental conditions related parameters were also found in the reported models, including the solubility of ENMs, aggregation of ENMs, and relevant aggregated ENM size in the media. The rest of the commonly identified descriptors by nano-(Q)SARS or read-across models are seen as representing the intrinsic properties of the metal oxides, and generally belong to three groups that address different aspects of the material eliciting adverse effects: descriptors describing the surface redox and catalytic properties of metal oxides; descriptors indicating the process of breaking of chemical bonds, detachment of ion and electron; and descriptors revealing the activity of ions released from ENMs.

**Figure 3:**
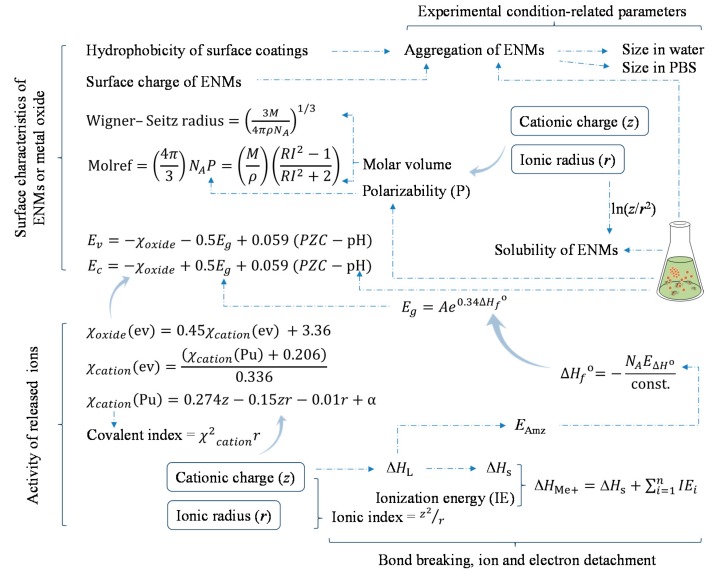
Profiling the toxicity of metal-based ENMs based on identified descriptors. Dashed line indicates the simplified (mutual) correlation between the descriptors. The descriptors are grouped as relating to the surface characteristics of ENMs or metal oxide, the activity of released ions, the bond breaking, ion and electron detachment, and the medium-related parameters. Molref, molar refractivity; *M*, molecular weight; *ρ*, density; *N*_A_, Avogadro’s number; RI, refractive index; PZC, point of zero charge; *E*_v_, valence band energy; *E*_c_, conduction band energy; *E*_g_, band gap; χ_oxide_, electronegativity of metal oxide; χ_cation_, electronegativity of cation; *E*_Amz_, atomization energy; ∆*H*_L_, lattice energy; ∆*H*_s_, enthalpy of sublimation; ∆*H*_Me+_, enthalpy of formation of a gaseous cation having the same oxidation state as that in the metal oxide structure; ∆*H_f_*^0^, enthalpy of formation of metal oxide nanocluster representing a fragment of the surface; *E*_∆*H*_^0^, energy associated with a single metal-oxygen bond in the metal oxide; PBS, phosphate buffered saline.

For the sake of conciseness, a simplified explanation of the correlations of these descriptors is depicted in Figure 3. The conduction and valence band energies of metal oxide can be derived from their electronegativity, energy gap, point of zero charge, and pH of the media; the electronegativity of a metal oxide is originated from the electronegativity of the corresponding cation, which can be determined by the cationic charge and ionic radius based on the equations described in Figure 3 [19]. The cationic charge and ionic radius likewise relate to the properties of metal oxides such as ionization energy [61], ionic index and atomization energy [21], lattice energy [28], enthalpy of sublimation [21,28], and the enthalpy of formation of a gaseous cation having the same oxidation state as in the metal oxide structure [28]. Additionally, the cationic charge and ionic radius also relate to the polarizability and molar volume of the metal oxide [40], and subsequently other properties which are associated with these descriptors such as molar refractivity [62] and Wigner–Seitz radius [33]. Burello [63] also classified the solubility of metal oxide ENMs in water and acidic media using the cationic charge and ionic radius. Therefore, it seems like the metal oxide ENMs which are able to release ions with smaller charge and larger ionic radius could induce higher toxicity to biota. That is to say, in general, within the same group of the periodic table, the larger the period that a metal belongs to (thus bigger atomic radius) the higher is the toxicity for the metal oxide ENMs formed by that metal; and within the same period in the table, metals on the left (thus smaller cationic charge) tend to form ENMs with higher toxicity compared with metals on the right. Meanwhile, metal oxide ENMs with low-valent metals may induce higher toxicity compared with ENMs composed of the same metal but of higher-valence. This corresponds with the study reported by Mu et al. [35] which predicted the toxicity of 51 metal oxide ENMs to *Escherichia coli* (presented in a periodic table).

It is commonly indicated that the release of ions and generation of reactive oxygen species (ROS) are two of the main mechanisms of metallic ENMs triggering toxicity, besides the possible direct steric hindrance caused by the particles per se and the ENMs acting as carriers of toxic chemicals (described as the Trojan-horse mechanism). In fact, both the detachment of ions or electrons from an ENM surface could lead to the formation of ROS. For instance, according to the Haber–Weiss–Fenon cycle [22,64], Cu^2+^ could act as a catalyst for the formation of hydroxyl radicals (OH^●^), which subsequently leads to the generation of superoxide anion radicals ($O_{2}^{●-}$):

(2) $$O_{2}^{●-}+{\mathrm{Cu}}^{2+}\to O_{2}+{\mathrm{Cu}}^{+}$$

(3) $${\mathrm{Cu}}^{+}+H_{2}O_{2}\to {\mathrm{Cu}}^{2+}+{\mathrm{OH}}^{-}+{\mathrm{OH}}^{●}$$

(4) $$O_{2}+e\to O_{2}^{●-}$$

Meanwhile, the detachment of an electron from the surface of TiO_2_ ENMs (which could be activated by solar radiation) is also able to initiate a series of reactions leading to the formation of OH^●^ and $O_{2}^{●-}$ [36]:

(5) $${\mathrm{TiO}}_{2}\overset{hv}{\Rightarrow }{\mathrm{TiO}}_{2}^{+}+\bar{e}$$

(6) $$\bar{e}+O_{2}\to O_{2}^{●-}$$

(7) $$O_{2}^{●-}+2H^{+}+\bar{e}\to H_{2}O_{2}$$

(8) $$O_{2}^{●-}+H_{2}O_{2}\to {\mathrm{OH}}^{●}+{\mathrm{OH}}^{-}+O_{2}$$

(9) $$H^{+}+H_{2}O\Rightarrow {\mathrm{OH}}^{●}+H^{+}$$

The generation of these ROS will disturb the cellular balance between the levels of oxidized and reduced species, and consequently provoke oxidative stress in cells [22]. Thus, the intrinsic properties of a metal oxide (e.g., cationic charge and ionic radius) which are of significant importance for the possibility of electron transfer, bond breaking, and release of ions, seem to play a pivotal role in affecting the toxicity of ENMs. This is why doubt has arisen about whether the toxicity of metallic ENMs is nano-specific or comparable with that of corresponding dissolvable materials [65,66,67]. However, undoubtedly, the other above-identified factors such as ENM surface chemistry, solubility of ENMs, and the experimental conditions are certainly able to alter the biological activity of metallic ENMs, by directly modifying the toxicity of the materials or by changing the bioavailability of ENMs for different species or cells [24]. In the study of Zhang et al. [19], the solubility of metal oxide ENMs is one of the discriminating factors for classifying the observed toxicity. Solubility successfully explained the high toxicity of CuO and ZnO ENMs as the conduction band energy of the two ENMs has no overlap with the cellular redox potential (−4.12 to −4.84 eV). Observations of the nanotoxicity affected by ENMs shape were thereupon reported for ZnO nanospheres, nanosticks, and cuboidal submicron particles [68]. The needle-shaped ZnO NPs were proven to be more toxic to *Phaeodactylum tricornutum* than other morphologically different NPs with equal solubility and ion release [69]. Therefore, it seems that whether the toxicity induced by metallic ENMs should be considered as nano-specific is case-dependent.

Recently, a categorization framework of ENMs called the decision-making framework for the grouping and testing of nanomaterials (DF4nanoGrouping) was proposed based on the intrinsic material properties, system-dependent properties, and in vitro and in vivo effects of ENMs [70]. This framework assigns ENMs into four main groups (MG) and determines to what extent the ENMs needs to be further evaluated. Specially, ENMs in MG 1 (soluble ENMs) are suggested to be handled by the read-across of the properties of dissolved materials from the bulk counterparts; ENMs in MG 4 (active ENMs) are advised to be carefully evaluated and merit in-depth investigations in light of the risk assessment. ENMs in MG4 are for instance CeO_2_ ENM-211, CeO_2_ ENM-212, TiO_2_ ENM-105, SiO_2_ ENM·acrylate, and SiO_2_ ENM·phosphate [71]. Thus, based on this grouping strategy, the requirement on structural information of ENMs can be waived for the materials of MG 1. This kind of data is on the other hand of crucial importance for the “active” ENMs (Main Group 4), for the purpose of calculating nano-specific descriptors in case of generating in silico models for ENMs and for the purpose of grouping ENMs based on different properties in case of developing SSDs to diminish variabilities and levels of uncertainties.

## 5. Outlook

As previously addressed, one of the most fundamental issues in developing in silico models for the hazard assessment of ENMs is the availability and quality of laboratory derived data. For further experimental studies on nanotoxicity, providing comprehensive information according to standardized test protocols is of vital importance, together with widely accepted evaluation criteria for data quality. Meanwhile, maximizing the use of existing information seems realistic, practical, and favorable for this new frontier. One suggestion for this purpose is to transfer toxicity data between different endpoints with suitable assessment factors, which has been proven as a feasible way to obtain needed data given very limited available information. For example, in the study of Wang et al. [47,50], an assessment factor of 10 was used to transfer LC/EC25–50 to no observed effect concentrations; a factor of 2 for the LC/EC10–20; and a factor of 1 for other endpoints such as LOEC, LED, MIC, HONEC, and NOEC. Likewise, this solution was also employed in different studies to overcome the problem of data scarcity [49,51,57]. Even though uncertainty in doing so still remains debatable, this may be one of the most pragmatic ways of facing the current challenges of lack of toxicity data.

**Figure 4:**
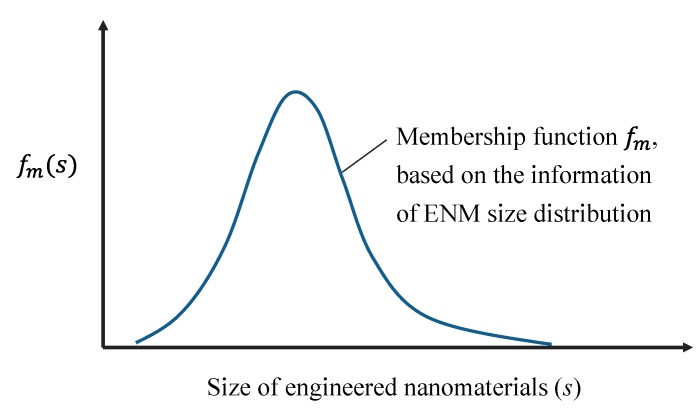
An explanation of considering the fuzzy set theory in handling the heterogeneity of ENM size for the computation of nano-specific descriptors.

The structural complexity of ENMs has brought difficulty to computationally characterize the structure of ENMs in a comprehensive way. The incorporation of size information of ENMs into computational parameters also faces obstacles. An attempt to overcome this challenge is the study of Tämm et al. [72] in which a set of novel, theoretical size-dependent nano-descriptors for ENMs was developed. However, the key problem is that the size of ENMs in reality is never a fixed value but rather a distribution of sizes. Preparing 100% homogeneous ENMs also does not seem possible in the near future. One proposed idea here is to adapt the calculation of nano-descriptors by combining them with fuzzy set theory. The fuzzy set theory permits the gradual assessment of the membership of elements in a set, instead of assigning an element into either one set or another [73]. Similarly, an ENM normally has a size distribution ranging, for example, 10–30 nm rather than a homogeneous size of 20 nm. Thus, if a descriptor (*D*_n_) for a cluster of an ENM of size (*s*) can be expressed as:

(10) $$D_{n}=f\left(s\right)$$

then the calculation of descriptors combined with fuzzy set theory ($D_{n}^{\prime }$) can be described as:

(11) $$D_{n}^{\prime }=\sum^{\text{​}}f_{m}\left(s\right)f\left(s\right)$$

when *s* is a discrete variable in f(s), or

(12) $$D_{n}^{\prime }=\int_{a}^{b}f_{m}\left(s\right)f\left(s\right)d_{s},\;a\leq s\leq b$$

when *s* is a continuous variable in f(s); $f_{m}$ is the membership function extracted from the information on the ENM size distribution (see Figure 4).

Another issue worth mentioning relates to the linking of structural characteristics of ENMs with their biological activities. As shown in Table 1, even though some of the studies constructed models solely based on theoretical descriptors, the experimental descriptors such as zeta potential, concentration of ENMs, aggregation parameter, size in media, etc. were also incorporated in other models. This agrees with the well-known fact that the dynamic transformation of ENMs in media is able to alter the biological profiles of the materials. Thus in some cases toxicity information of ENMs can be poorly modeled without considering this transformation. However, dilemma situations arise as the safe-by-design approach of ENMs tends to favor the information of ENM safety purely based on their structures. For the next step, modeling and prediction of ENM behavior and transformation in different media (e.g., aggregation) could be considered based on ENM structural characteristics; and also the link of transformed characteristics of ENMs in the media to relevant biological activity. Different dose metrics in expressing the effective dose should be also taken into account for the modeling [74]. Mass should not be the sole option in this context as nanotoxicity is influenced by many different physicochemical properties of ENMs [75].

**Figure 5:**
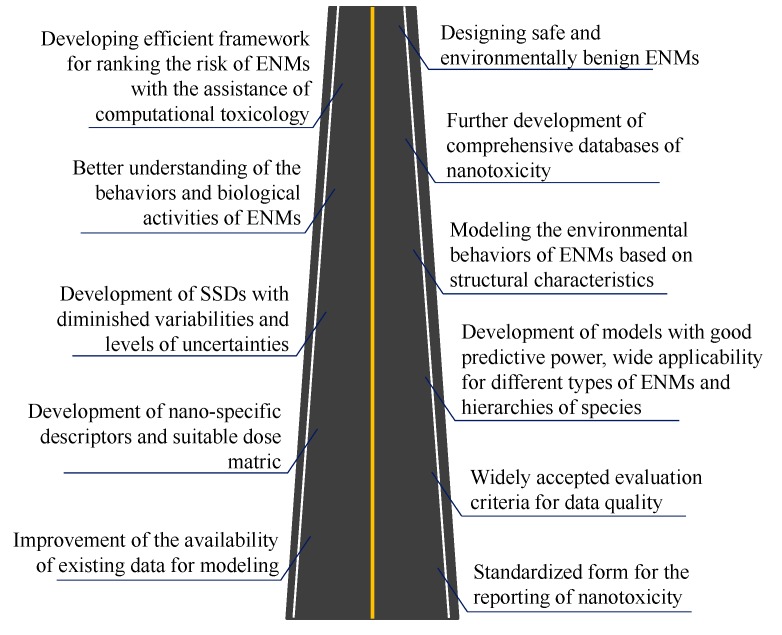
A roadmap indicating the future milestones of using computational toxicology in assisting the hazard assessment of ENMs.

In the near future, the first milestone to be achieved regarding the use of computational toxicology in hazard assessment of ENMs should be a standardized form for reporting nanotoxicity (see Figure 5). Maximizing the use of existing data of nanotoxicity should also be considered. Setting up widely accepted criteria is crucial for evaluating the quality of laboratory derived data for both existing and newly reported data. Development of novel nano-specific descriptors and incorporation of proper dose metrics are needed when performing modeling. The newly constructed nano-(Q)SARs and read-across models based on data with improved quality and availability are expected to have improved predictive power with broader applicability (suited for more types of ENMs and wider spectrum of species). The SSDs for deriving the maximum acceptable concentrations of ENMs are also expected to have diminished variabilities and levels of uncertainties. Meanwhile, linking the structural characteristics of ENMs to their environmental behavior and transformation is of great interest. Such work will provide further insight into the mechanisms underlying the biological profiles and environmental behavior of ENMs. In time, based on standardized criteria for reporting and evaluating nanotoxicity data, relevant databases with comprehensive information of all aspects will be developed. Upon these advances, construction of the framework ranking ENM hazard and associated risk aided by computational toxicology will highly contribute to the safe handling of ENMs and regulatory activities. Designing safe and environmentally benign ENMs supported by computational toxicology will also greatly benefit the minimization of risks brought by newly developed ENMs and the fast development of nanotechnology.

## 6. Conclusions

In conclusion, the added value of this review can be summarized as the following.

(i) An overview is provided of the current advances towards the development of in silico predictive models and SSDs for metallic ENMs. Based on reported models, factors such as solubility, hydrophobicity of ENM surface coating, and polarizability were concluded as enhancing the toxicity elicited by metallic ENMs. Meanwhile factors such as conduction band energy, ionization energy, and cationic charge were shown to play an opposite role in this respect. The studies on SSDs for ENMs showed that marginal risks are associated with the presence of Ag, TiO_2_, and ZnO ENMs in surface water, whereas high environmental risks are foreseen for those ENMs in sewage treatment effluents.
(ii) A proposal is presented for preparation of a thoroughly curated dataset related to reporting of future results of laboratory studies, in light of enclosing sufficient information to allow for optimal ENM-related modeling based on laboratory assays.
(iii) The mechanism of biological activities of metal-based ENMs is profiled based on employed descriptors. The intrinsic properties of ENMs such as cationic charge and ionic radius are considered pivotal in affecting nanotoxicity. However, surface chemistry of ENMs is shown to also be able to significantly modify the toxicity or bioavailability of metallic ENMs.
(iv) Several suggestions for further studies are provided in the outlook, with regard to the use of existing nanotoxicity data for modeling, computation of nano-specific descriptors, and consideration of the transformation of ENMs in media into modeling. Finally, a roadmap is depicted to optimize the use of computational toxicology in hazard assessment of ENMs and to further advance the broader field of ENM-related modeling.

## Figures and Tables

**Table 1:** Summary of the state-of-the-art of developed (Q)SARs and read across approaches for metal-based engineered nanomaterials (ENMs).

Reference	Indicated ENM Characteristics in Models	Theoretical Descriptor	Experimental Descriptor	ENMs	Tested Organism	Data Retrieved from
[18] *	Number of metal and oxygen atoms, molecular weight, atomization energy, group and period in the periodic table, size, isoelectric point, zeta potential, concentration	√	√	9 metal oxide ENMs	BEAS-2B cells	N/A
[19]	Band gap energy, overlap of conduction band energy levels with the cellular redox potential (−4.12 to −4.84 eV), solubility	√	√	24 metal oxide ENMs	BEAS-2B cells, RAW 264.7 cells	N/A
[20]	Mass density, molecular weight, aligned electronegativity, covalent index, cation polarizing power, Wigner–Seitz radius, surface area, surface-area-to-volume ratio, aggregation parameter, two-atomic descriptor of van der Waals interactions, tri-atomic descriptor of atomic charges, tetra-atomic descriptor of atomic charges, size in DMEM	√	√			[19]
[21] *	Atomization energy, atomic mass, size, conduction band energy, metal oxide ionization energy, electronegativity, ionic index of metal cation	√				
[22]	Enthalpy of formation of metal oxide nanocluster representing a fragment of the surface, Mulliken’s electronegativity of the cluster	√		18 metal oxide ENMs	HaCaT cells	N/A
[23] *	Molar volume, polarizability, size	√	√	41 metallic ENMs	Mammalian cells	Multiple resources
[24] *	Size, relaxivities, zeta potential	√	√	50 metallic ENMs	Endothelial cells, vascular smooth muscle cells, human HepG2 cells, RAW 264.7 cells	[25]
[26]	Indicator variables of core material, surface coating, and surface charge	√				
[27]	(i) Size, relaxivities, zeta potential; (ii) Oxygen percent, molar refractivity, polar surface area	√	√	(i) 44; (ii) 17 metallic ENMs	(i) Endothelial cells, vascular smooth muscle cells, human HepG2 cells, RAW 264.7 cells; (ii) *E. coli*	[25,28]
[29] *	Size, concentration, size in phosphate buffered saline, size in water, zeta potential	√	√	24 TiO_2_, 18 ZnO ENMs	Rat L2 lung epithelial cells, rat lung alveolar macrophages	N/A
[30]	Size, concentration, size in phosphate buffered saline, size in water	√	√			[29]
[31]	Molecular weight, cationic charge, mass percentage of metal elements, size, aggregation size	√	√	(i) 17; (ii) 18 metal oxide ENMs	(i) *E. coli*; (ii) HaCaT cells	[22,28]
[32] *	Enthalpy of formation of a gaseous cation, Mulliken’s electronegativity of the cluster	√				
[33]	(i) *S*_1_, Wigner–Seitz radius, mass density, cation polarizing power, *S*_2_, *S*_3_, proportion of surface molecules to molecules in volume; (ii) *S*_1_, Wigner–Seitz radius of oxide’s molecule, mass density, covalent index of the metal ion, *S*_2_, aggregation parameter	√	√			
[34]	Enthalpy of formation of a gaseous cation, enthalpy of formation of metal oxide nanocluster representing a fragment of the surface, Mulliken’s electronegativity of the cluster	√				
[28]	Enthalpy of formation of a gaseous cation	√		17 metal oxide ENMs	*E. coli*	N/A
[35]	Polarization force, enthalpy of formation of a gaseous cation	√				[28]
[36]	Charge of the metal cation corresponding to a given oxide, metal electronegativity	√				
[37]	Dark: absolute electronegativity of the metal and the metal oxide; Light: molar heat capacity, average of the alpha and beta LUMO (lowest unoccupied molecular orbital) energies of the metal oxide	√				N/A
[15] *	Molecular polarizability, accessible surface area, solubility	√		400; 450; 166 metallic ENMs	Various species	[38]; OCHEM
[16] *	Molar volume, electronegativity, polarizability, size, hydrophobicity, polar surface area	√	√	229 metallic ENMs	Various species	Multiple resources
[17]	Concentration, shell composition, surface functional groups, purity, core structure, and surface charge	√	√	82 ENMs including metal and metal oxide ENMs, dendrimer, polymeric etc.	Zebrafish embryo	NBI knowledgebase

Classification models are marked separately by means of an asterisk (*). N/A indicates that relevant information is not available. *E coli*, *Escherichia coli*; BEAS-2B, transformed bronchial epithelial cells; RAW 264.7, murine myeloid cells; HaCaT, human keratinocyte cells; HepG2 cells, hepatocytes; *S*_1_, unbonded two-atomic fragments [Me]···[Me], which were encoded based on SiRMS-derived descriptors, describing the distance where potential reaches minimum at van der Waals interactions; *S*_2_, SiRMS-derived number of oxygen’s atoms in a molecule, which was described by their electronegativity; *S*_3_, tri-atomic fragments [Me]-[O]-[Me], which were encoded by SiRMS-derived descriptors, encoding electronegativity; OCHEM, Online chemical modeling environment [39]; NBI Knowledgebase, Nanomaterial-Biological Interactions Knowledgebase (available online http://nbi.oregonstate.edu/).

**Table 2:** Summary of the state-of-the-art of the developed SSDs for metal and metal oxide ENMs. N/A indicates that relevant information is not available.

Reference	Type of ENMs	Reported HC5s	Number of Species in SSDs	Environmental Compartment
Jacobs et al., 2016 [46]	TiO_2_	N/A	31	Water
Wang et al., 2016 [47]	FeO_x_	0.218 (0.169–0.267) mg/L, 15–85% percentiles	12	Water
Kwak et al., 2016 [48]	Ag	0.03173 mg/L (acute toxicity); 0.000614 mg/L (chronic toxicity)	8 (acute toxicity); 5 (chronic toxicity)	Water
Coll et al., 2016 [49]	(i) Ag; (ii) TiO_2_; (iii) ZnO	(i) 0.000017 (0.000014–0.000021) mg/L in freshwater, 8.2 (4.3–12.5) mg/kg in soil; (ii) 0.0157 (0.0106–0.0207) mg/L in fresh water, 91.1 (47.6–134.9) mg/kg in soil; (iii) 0.001 (0.0006–0.00138) mg/L in freshwater, 1.1 (0.6–1.6) mg/kg in soil, 95% confidence intervals	(i) 33 (water), 4 (soil); (ii) 31 (water), 2 (soil); (iii) 21 (water), 7 (soil)	Water, soil
Wang et al., 2016 [50]	Silica	1.023 (0.787–1.265) mg/L, 15–85% percentiles	8	Water
Mahapatra et al., 2015 [51]	Au	N/A	8 (water)	Water, soil
Semenzin et al., 2015 [52]	TiO_2_	0.02 mg/L	34	Water
Adam et al., 2015 [53]	(i) ZnO; (ii) CuO	(i) 0.07 (0.04–0.19) mg/L; (ii) 0.19 (0.06–0.59) mg/L, 90% confidence intervals	(i) 12; (ii) 13	Water
Garner et al., 2015 [45]	(i) Ag; (ii) Cu; (iii) CuO; (iv) ZnO; (v) Al_2_O_3_; (vi) CeO_2_; (vii) TiO_2_	N/A	(i) Uncoated-Ag: 8, PVP-Ag: 6; (ii) 4; (iii) 5; (iv) 7; (v) 9; (vi) 7; (vii) 8	Water
Nam et al., 2015 [54]	Au	0.29 mg/L	7	Water
Botha et al., 2015 [55]	Au	42.78 mg/L	4	Water
Haulik et al., 2015 [56]	(i) Ag; (ii) TiO_2_; (iii) ZnO	(i) 0.00015; (ii) 0.275; (iii) 3.246 mg/L	(i) 14; (ii) 11; (iii) 10	Water
Gottschalk et al., 2013 [57]	(i) Ag; (ii) TiO_2_; (iii) ZnO	(i) 0.00001; (ii) 0.06151; (iii) 0.00985 mg/L	(i) 12; (ii) 18; (iii) 17	Water
Chen et al., 2017 [58]	(i) Ag; (ii) CuO; (iii) ZnO; (iv) CeO_2_; (v) TiO_2_	HC5s were calculated for various SSDs	Different hierarchies of species were used	Water
